# A Novel Deep Learning Model for Breast Tumor Ultrasound Image Classification with Lesion Region Perception

**DOI:** 10.3390/curroncol31090374

**Published:** 2024-08-28

**Authors:** Jinzhu Wei, Haoyang Zhang, Jiang Xie

**Affiliations:** 1 School of Medicine, Shanghai University, Shanghai 200444, China; jinzhuwei@shu.edu.cn; 2 School of Computer Engineering and Science, Shanghai University, Shanghai 200444, China; zhy-jsj-0105@shu.edu.cn

**Keywords:** multi-feature fusion, breast tumor classification, attention mechanisms, multi-task learning

## Abstract

Multi-task learning (MTL) methods are widely applied in breast imaging for lesion area perception and classification to assist in breast cancer diagnosis and personalized treatment. A typical paradigm of MTL is the shared-backbone network architecture, which can lead to information sharing conflicts and result in the decline or even failure of the main task’s performance. Therefore, extracting richer lesion features and alleviating information-sharing conflicts has become a significant challenge for breast cancer classification. This study proposes a novel Multi-Feature Fusion Multi-Task (MFFMT) model to effectively address this issue. Firstly, in order to better capture the local and global feature relationships of lesion areas, a Contextual Lesion Enhancement Perception (CLEP) module is designed, which integrates channel attention mechanisms with detailed spatial positional information to extract more comprehensive lesion feature information. Secondly, a novel Multi-Feature Fusion (MFF) module is presented. The MFF module effectively extracts differential features that distinguish between lesion-specific characteristics and the semantic features used for tumor classification, and enhances the common feature information of them as well. Experimental results on two public breast ultrasound imaging datasets validate the effectiveness of our proposed method. Additionally, a comprehensive study on the impact of various factors on the model’s performance is conducted to gain a deeper understanding of the working mechanism of the proposed framework.

## 1. Introduction

Breast cancer is the most common malignant tumor among women and a leading cause of cancer-related death worldwide [1,2]. Over the past four decades, the incidence of breast cancer has been rising. According to the latest data from the International Agency for Research on Cancer of the World Health Organization, there were approximately 2.3 million new cases and 660,000 deaths in 2022 globally. It is estimated that by 2045 there will be about 3.3 million new cases and 1 million deaths due to breast cancer. Clinically, the strategy for breast cancer is mainly “early diagnosis and early treatment”, as the timing of diagnosis and treatment is directly linked to prognosis. Therefore, finding suitable diagnostic and therapeutic strategies is crucial. Early diagnosis plays a key role in controlling the progression of breast cancer.

With the advancement of medical imaging technology, the use of imaging exams for cancer detection, early diagnosis, and assessment of therapeutic efficacy has contributed to improving early detection rates of breast cancer and reducing mortality rates among breast cancer patients. Currently, early clinical screening assessments for breast cancer include ultrasound, computed tomography (CT), mammography, magnetic resonance imaging (MRI), positron emission tomography (PET), and other medical imaging techniques. Compared to other imaging methods, ultrasound has characteristics such as rapid and convenient operation, no radiation, and low cost, making it the most effective means for promoting widespread and quick breast tumor screening. However, ultrasound examination heavily relies on the diagnostic level of the radiologist, with differences in analysis between different doctors and disagreements in interpretation during diagnosis. Additionally, manual analysis of a large number of breast ultrasound screening images is a time-consuming task that significantly increases the overall workload of radiologists. The inherent characteristics of breast ultrasound images also bring additional challenges to image analysis, such as the similarity of texture and contrast between the lesions and surrounding tissues, heterogeneity of lesion tissue, and user dependence on scan quality [3]. Thus, interpretation of many cases can lead to inaccurate diagnoses, particularly for radiologists with less experience [4].

To address these challenges and promote clinical applications such as localization and classification [5,6,7,8], computer-aided diagnosis (CAD) systems have been developed. The goal of lesion classification is to stratify lesions into different subtypes to support appropriate treatment plans, while lesion localization and segmentation can facilitate classification tasks to achieve better diagnostic accuracy [9,10,11]. Unlike the approach of handling these tasks separately, multi-task learning (MTL) techniques are designed to jointly perform lesion segmentation and classification [11,12,13]. A typical MTL model adopts a network architecture that includes a shared feature extractor and two task-specific branches for lesion segmentation and classification, respectively. By using a mixed task-related loss function for training, this shared trunk model can improve classification performance and robustness. The network design and training process allow for information sharing between tasks, thereby reducing the risk of model overfitting.

However, training MTL models is significantly more complex and challenging compared to single-task models. The potential for information-sharing conflicts between different tasks not only increases the complexity of the model but can also degrade the performance of the main task. Balancing cross-task information sharing is crucial to ensuring the performance of the main task [14]. To address this issue, several methods have been proposed, such as task-specific loss weighting [15] and gradient modulation [16], which aim to balance the contribution of each task to the combined loss.

In recent years, many studies have employed the attention mechanism to address information sharing conflicts in multi-task learning, particularly in the segmentation and classification of ultrasound images [17,18]. The attention module highlights key input features and attenuates secondary features [15], generating task-specific representations that reduce interference during model optimization. Additionally, the attention mechanism integrates multi-scale features to capture both local and global information, thereby enhancing lesion segmentation performance [19,20,21]. Current research on joint segmentation tasks primarily focuses on aiding breast tumor classification. However, this requires pixel-level segmentation annotation, which demands substantial time and human resources. Moreover, pixel-level segmentation annotation often involves subjective judgments, potentially leading to inconsistencies. Different annotators may provide varying annotations, which can result in inconsistent dataset quality. Additionally, current research mainly concentrates on designing loss functions to enhance the performance of the classification task, with less emphasis on how the knowledge learned from auxiliary tasks can provide critical tumor feature information for the breast tumor classification task.

In this study, to address the aforementioned issues, we propose a novel Multi-Feature Fusion Multi-Task (MFFMT) convolutional neural network model for joint lesion region perception and benign–malignant classification of breast tumors, effectively enhancing classification performance. The model first extracts and integrates multi-scale features through a Contextual Lesion Enhancement Perception (CLEP) module, obtaining richer contextual feature information about breast tumor lesions. Then, these lesion features are effectively fused with the semantic features used for classification through the Multi-Feature Fusion (MFF) module, significantly improving the performance of the benign–malignant classification of breast tumors. The experimental results demonstrate the effectiveness of our proposed method, as validated by experiments conducted on two BUS image datasets. Additionally, we explore the impact of various network parameters to gain a deeper understanding of the distinctive features of our design framework. The primary contributions of this study are as follows:

- Information sharing and conflict reduction: In the context of multi-task learning for medical imaging, conflicting gradients and insufficient information sharing between tasks can hinder performance. Our MFFMT model directly addresses these issues by employing an adaptive Multi-Feature Fusion mechanism that ensures complementary features are shared while reducing task interference. This approach not only enhances classification accuracy but also tackles one of the persistent challenges in multi-task learning.
- Enhanced lesion perception: The challenge of accurately detecting lesions in ultrasound images due to factors like noise, low contrast, and varying lesion sizes is well known. Our Contextual Lesion Enhancement Perception (CLEP) module is specifically designed to overcome these limitations. By capturing multi-scale and location-specific features, the module strengthens the model’s focus on the lesion area, improving overall diagnostic reliability.
- Feature integration for noise reduction: Ultrasound images often suffer from noise and limited contrast, which can obscure critical features relevant to classification. Our Multi-Feature Fusion (MFF) module mitigates these issues by integrating features from different tasks, thus highlighting tumor-specific characteristics while suppressing irrelevant information. This integration is key to improving classification outcomes, especially in noisy or low-contrast imaging environments.
- Application potential and clinical significance: The practical applicability of models in clinical settings is often limited by generalization issues when tested on diverse datasets. We conducted extensive evaluations on two publicly available breast tumor ultrasound datasets, demonstrating that our model not only addresses the aforementioned technical challenges but also shows significant promise for clinical deployment, contributing to more accurate and consistent tumor classification.

## 2. Materials and Methods

### 2.1. Breast Tumor Dataset

**Figure 1:**
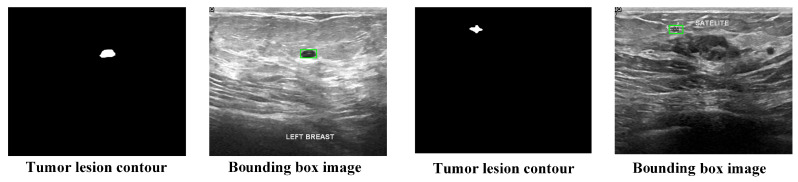
Example of BUSI dataset preprocessing.

This study assesses the model on two publicly available breast ultrasound image datasets. The first dataset is the Breast Ultrasound Image (BUSI) dataset, which is a publicly available resource provided by Al-Dhabyani et al. [22]. The database contains 780 images from 600 patients, with 133 images showing normal, non-tumor tissue and the remaining 647 containing tumor images. Each image includes labeled lesion segmentation contours and lesion type. Since the BUSI dataset only provides lesion segmentation contours and lacks bounding box annotations, we generate the bounding boxes for BUSI images during data preprocessing based on the lesion segmentation contours. An example of a preprocessed image is shown in Figure 1. It is noteworthy that some images in the dataset contain two or more tumors, so when generating bounding boxes, multiple bounding box annotations are created for each image, making each bounding box an individual data sample. Table 1 presents the detailed information of the preprocessed dataset for training and testing the model.

The second dataset, MIBUS, is a publicly available breast lesion ultrasound video dataset released by Zhu Lei et al., consisting of 188 videos. This dataset includes 113 malignant videos and 75 benign videos [23]. The 188 videos contain a total of 25,272 images, with the number of ultrasound images per video ranging from 28 to 413. Each video provides a complete scan of the tumor, from its appearance to its maximum size and eventual disappearance. All videos were recorded using LOGIQ-E9 (GE HealthCare, Chicago, IL, USA) and PHILIPS TIS L9-3 (Philips, Amsterdam, The Netherlands). A rectangular region around the breast lesion is annotated in each video frame, and the lesion in the video is assigned a corresponding classification label.

First, 38 videos were randomly and uniformly selected from the dataset to be used as the test set (approximately 20% of the dataset), while the remaining videos were designated as the training set. This approach ensures that images from the same patient do not appear in both the training set and the test set. Then, from this split video dataset, 5 to 15 images were selected from each ultrasound video as samples for final model training and testing. Table 2 provides detailed information about the MIBUS dataset used for model training and testing.

### 2.2. MFFMT Model Architecture

**Figure 2:**
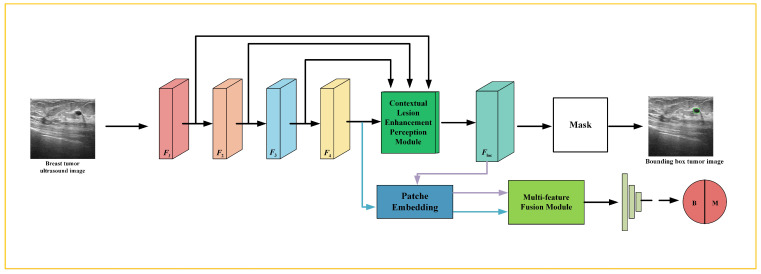
The Multi-Feature Fusion Multi-Task model (MFFMT) architecture diagram.

This section presents the architecture of the MFFMT model. As shown in Figure 2, the MFFMT model combines ResNet as its feature extractor (FE) to extract features at different levels from breast tumor ultrasound images. Additionally, the model includes a Contextual Lesion Enhancement Perception module for perceiving the tumor lesion area, enabling the localization of the tumor region. Lastly, for breast tumor classification, the model employs a Multi-Feature Fusion module to integrate different tumor-related features, which are then input into the classifier for classification.

Given breast tumor ultrasound images, ResNet extracts multi-scale feature maps, capturing both local and global information. These feature maps are then fed into the CLEP module for lesion region perception. The lesion enhancement perception feature map $F_{\mathrm{loc}}$, obtained from the CLEP module, is further utilized in the breast tumor classification task to improve the performance of lesion type classification. Notably, feature fusion explicitly associates the classification and lesion perception branches through a module that takes ResNet’s feature map $F_{4}$, and CLEP’s feature map $F_{\mathrm{loc}}$ as inputs. This design alleviates potential conflicts and ensures enhanced performance for the breast tumor classification task. The following sections provide a detailed discussion of each module.

#### 2.2.1. Feature Extractor

The feature extractor (FE) is designed in a hierarchical structure, comprising a series of *n* convolutional blocks. Given a two-dimensional breast tumor ultrasound image $X\in R^{M\times N}$, the FE extracts multi-scale feature maps $F_{1},F_{2},F_{3},$ and $F_{4}$ using each convolutional block. These extracted feature maps serve as the inputs to the CLEP module, while only the top-level extracted feature map $F_{4}$ is used as the input to the classification branch. The FE can be described as follows:

(1) $$\begin{array}{c} \left(F_{1},F_{2},F_{3},F_{4}\right)=F\left(X,\Theta_{F}\right) \end{array}$$

where *F* represents the mapping function of the FE parameterized by $\Theta_{F}$.

#### 2.2.2. Contextual Lesion Enhancement Perception (CLEP) Module

**Figure 3:**
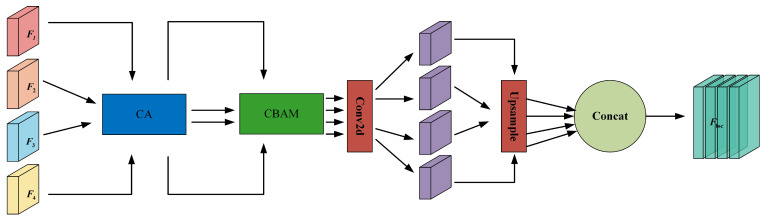
Schematic diagram of the CLEP module.

As shown in Figure 3, the CLEP module incorporates a Coordinate Attention (CA) module, a Convolutional Block Attention Module (CBAM), and a feature fusion operation. This design is motivated by the versatility of CBAM when integrated into various CNNs, which can seamlessly enhance both classification and localization performance [24]. However, CBAM only captures local relationships and cannot model the long-range dependencies crucial for visual tasks. Therefore, we introduce a novel Coordinate Attention (CA) module on top of CBAM, embedding positional information into channel attention. This enables the network to capture directionally aware and position-sensitive information, facilitating the model’s ability to recognize targets of interest [25].

**Figure 4:**
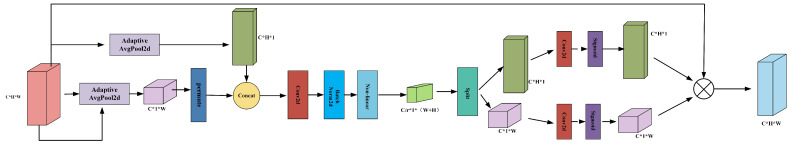
Schematic diagram of the Coordinate Attention module (CA).

Specifically, the multi-scale feature maps $F_{1},F_{2},F_{3},F_{4}$ extracted from the FE are initially fed into the CA module, as illustrated in Figure 4. This module encodes both channel relationships and long-range dependencies through two steps: coordinate information embedding and coordinate attention generation.

Global average pooling is commonly used in channel attention to encode spatial information in a global manner. However, this approach compresses global spatial information into a single channel descriptor, making it difficult to retain positional information, which is crucial for capturing spatial structure in visual tasks. To facilitate the attention block in accurately capturing long-range interactions in space, we decompose the global pooling and convert it into a pair of 1D feature encoding operations. Specifically, for a given feature *F*, we use two pooling kernels with spatial ranges of (H,1) or (1,W), encoding each channel along the horizontal and vertical coordinates, respectively. Therefore, the output for the *c*th channel at height *h* can be expressed as

(2) $$\begin{array}{c} \zeta_{c}^{h}\left(h\right)=\frac{1}{W}\sum_{i=0}^{W}F_{c}\left(h,i\right) \end{array}$$

Similarly, the output for the *c*th channel at width *w* can be written as

(3) $$\begin{array}{c} \zeta_{c}^{w}\left(w\right)=\frac{1}{H}\sum_{j=0}^{H}F_{c}\left(j,w\right) \end{array}$$

These two transformations aggregate features along two spatial dimensions, resulting in a pair of directionally aware feature maps. These transformations also allow our attention block to capture long-range dependencies in one spatial direction while preserving precise positional information in the other. This enables the network to more accurately localize objects of interest.

To leverage these expressive capabilities, the module incorporates a second transformation, referred to as the coordinate attention generation. Specifically, given the aggregated feature maps derived from Equations (2) and (3), they are concatenated, and then, fed into a shared 1 × 1 convolution transformation function $F_{1}$. This process generates a feature map *f*:

(4) $$\begin{array}{c} f=\delta (F_{1}\left(\left[\zeta^{h},\zeta^{w}\right]\right)) \end{array}$$

where [·,·] represents the concatenation operation along the spatial dimension, and δ denotes a nonlinear activation function. The resulting feature map $f\in R^{C/r\times (H+W)}$ serves as an intermediate representation, encoding spatial information in both the horizontal and vertical directions. Here, *r* is a reduction ratio that determines the size of the block. Next, we split *f* along the spatial dimension into two separate tensors: $f^{h}\in R^{C/r\times H}$ and $f^{w}\in R^{C/r\times W}$. Two 1 × 1 convolution transformations, $F_{h}$ and $F_{w}$, are used to convert $f^{h}$ and $f^{w}$ into tensors with the same number of channels as the input feature map *F*. This results in

(5) $$\begin{array}{c} g^{h}=\sigma \left(F_{h}\left(f^{h}\right)\right) \end{array}$$

(6) $$\begin{array}{c} g^{w}=\sigma \left(F_{w}\left(f^{w}\right)\right) \end{array}$$

where σ represents the sigmoid function. To minimize the computational complexity, we often use an appropriate reduction ratio *r* (e.g., 32) to reduce the number of channels in *f*. The outputs $g^{h}$ and $g^{w}$ are then expanded and used as attention weights. Ultimately, the output *Y* of the Coordinate Attention block can be expressed as

(7) $$\begin{array}{c} y_{c}\left(i,j\right)=F_{c}\left(i,j\right)\times g_{c}^{h}\left(i\right)\times g_{c}^{w}\left(j\right) \end{array}$$

**Figure 5:**
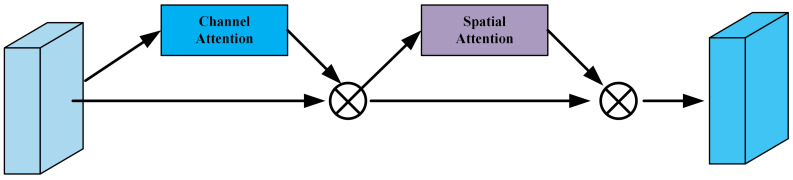
Schematic diagram of Convolution Block Attention Module (CBAM).

Then, the feature maps $F_{1},F_{2},F_{3},F_{4}$ processed by the Coordinate Attention (CA) module, yielding $F_{c1},F_{c2},F_{c3},F_{c4}$, are fed into the Convolutional Block Attention Module (CBAM). The CBAM module consists of a Channel Attention Module (CAM) and a Spatial Attention Module (SAM), which process the input feature maps sequentially, as shown in Figure 5. CBAM effectively compresses the input feature maps and selectively highlights the inter-channel and inter-spatial discriminative relationships in the input features.

Specifically, in the CBAM’s Channel Attention Module (CAM), the input feature map *F* undergoes parallel operations of a maximum pooling layer and an average pooling layer. This yields $F_{max}^{c}$ and $F_{avg}^{c}$, each producing a vector of dimensions C×1×1. These two vectors are then simultaneously processed by a multi-layer perceptron (MLP), consisting of an input layer, a hidden layer, and an output layer, with the respective weights denoted as $W_{0}$ and $W_{1}$. After passing through the MLP, the two vectors are merged by element-wise addition. Finally, a sigmoid activation function is applied, resulting in the final output of the Channel Attention Module. It can be represented as

(8) $$\begin{array}{cc} M_{C}\left(F\right) & =\sigma (\mathrm{MLP}(\mathrm{AvgPool}(F))+\mathrm{MLP}(\mathrm{MaxPool}(F)))\\ \; & =\sigma (W_{1}\left(W_{0}\left(F_{avg}^{c}\right)\right)+W_{1}\left(W_{0}\left(F_{max}^{c}\right)\right)) \end{array}$$

After the CAM, the output $M_{c}$ is a tensor of dimensions C×1×1. Then, element-wise multiplication is performed between $M_{c}$ and the corresponding channel of the original feature map, where the feature map has dimensions H×W. The result is a new feature map, denoted as $F_{C}$. In the Spatial Attention Module (SAM) of CBAM, the feature map $F_{C}$ undergoes two separate pooling operations on its channels: a max-pooling and an average-pooling operation. This results in a *C*-dimensional vector for each pooling operation, generating $F_{max}^{s}$ and $F_{avg}^{s}$, respectively. These two vectors are then concatenated and passed through a 7×7 convolution layer, followed by a sigmoid activation function, resulting in a 1×H×W feature map. This can be represented as

(9) $$\begin{array}{cc} M_{S}\left(F_{C}\right) & =\sigma (f^{7\times 7}\left(\left[F_{avg}^{s};F_{max}^{s}\right]\right))\\ \; & =\sigma (f^{7\times 7}\left(\left[AvgPool\left(F_{C}\right);MaxPool\left(F_{C}\right)\right]\right)) \end{array}$$

In a manner similar to the Channel Attention module, the final output $M_{S}\left(F_{C}\right)$ needs to be element-wise multiplied with the original input feature map $F_{C}$ to merge the two, producing the final output feature map for the CBAM module.

The feature maps processed by CA and CBAM are input into a convolutional layer to compress them into the same number of channels as the feature map $F_{2}$, thereby extracting the regions of interest (ROIs) related to the lesion. This refines the learned knowledge. Each compressed intermediate feature map is resized to match the dimensions of $F_{4}$, then concatenated into a merged feature map $F_{\mathrm{loc}}$. Finally, $F_{\mathrm{loc}}$ undergoes a series of convolutional layers, batch normalization (BN), and a sigmoid activation layer to produce the lesion perception feature map $F_{\mathrm{mask}}$, where each pixel represents the probability of being part of the lesion or background. A binarization function determines the predicted lesion position mask *Y*. Since the confidence threshold can impact the model’s final results, a higher threshold makes the model more conservative (i.e., reducing false positives), while a lower threshold makes the model more aggressive (i.e., capturing all possible lesions). Therefore, in this study, we adopt the commonly used default threshold of 0.5 as the confidence threshold.

(10) $$\begin{array}{c} P_{\mathrm{loc}}=D\left(F_{1},F_{2},F_{3},F_{4},\Theta_{D}\right) \end{array}$$

(11) $$\begin{array}{c} P_{\mathrm{mask}}=P_{\mathrm{pixel}}=\sigma \left(BN\left(\mathrm{Conv}2d\left(P_{\mathrm{loc}}\right)\right)\right) \end{array}$$

(12) $$\begin{array}{c} Y_{\mathrm{pixel}}=\mathrm{binarize}\left(P_{\mathrm{pixel}}>0.5\right) \end{array}$$

where σ denotes the sigmoid function, $P_{\mathrm{pixel}}$ represents the probability that each pixel in $P_{\mathrm{mask}}$ belongs to the lesion, *D* is the mapping function of CLEP, parameterized by the trainable parameters $\Theta_{D}$, and the binarize function is a threshold function.

#### 2.2.3. Multi-Feature Fusion Module

Subsequently, we use the Patch Embedding module to convert the feature maps $F_{4}$ and $F_{\mathrm{loc}}$ into a sequence of tokenized representations $F_{4}^{\mathrm{token}}$ and $F_{\mathrm{loc}}^{\mathrm{token}}$. The Patch Embedding process can be represented as

(13) $$\begin{array}{c} F_{4}^{\mathrm{token}}=PE\left(F_{4}\right) \end{array}$$

(14) $$\begin{array}{c} F_{\mathrm{loc}}^{\mathrm{token}}=PE\left(F_{\mathrm{loc}}\right) \end{array}$$

**Figure 6:**
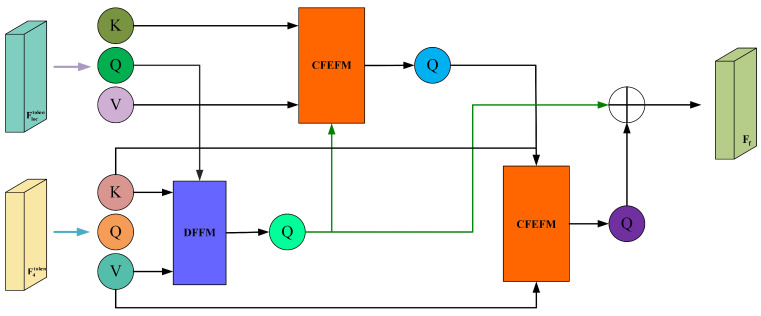
Schematic diagram of the Multi-Feature Fusion.

Next, the tokenized representations $F_{4}^{\mathrm{token}}$ and $F_{\mathrm{loc}}^{\mathrm{token}}$ are fed into the Multi-Feature Fusion (MFF) module to generate the fused feature $F_{f}$. The fusion process can be represented as

(15) $$\begin{array}{c} F_{f}=MFF\left(F_{4}^{\mathrm{token}},F_{\mathrm{loc}}^{\mathrm{token}}\right) \end{array}$$

where MFF consists of a Discrepancy Feature Fusion module and a pair of alternating Common Feature Enhancement Fusion modules, which are designed to extract global dependency features, namely, discrepancy features and common features. A schematic diagram of this fusion module is shown in Figure 6.

The goal of feature fusion is to obtain a composite feature map that captures prominent targets while preserving fine texture details. Therefore, leveraging the differences and shared features present in different feature maps is crucial for achieving optimal fusion performance. Inspired by the effectiveness of cross-attention mechanisms in extracting common features between images, we introduce the DFFM and the CFEFM.

**Figure 7:**
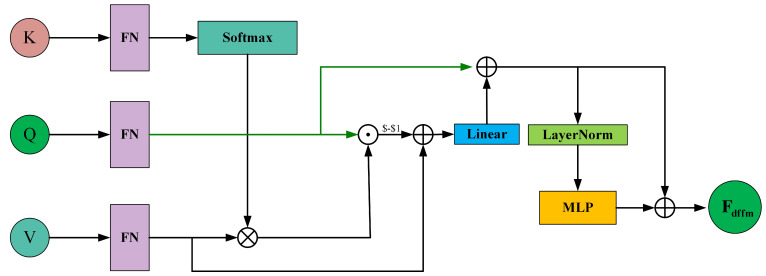
Schematic diagram of the Differential Feature Fusion Module (DFFM).

To effectively capture the differential features between $F_{4}$ and $F_{\mathrm{loc}}$ generated in the previous stage, we employ a DFFM in the form of cross-attention, as shown in Figure 7. It takes $F_{4}^{\mathrm{token}}$ and $F_{\mathrm{loc}}^{\mathrm{token}}$ as input and outputs features that highlight the differences.

Specifically, to explore the long-distance relationships of the feature $F_{loc}$, we partition $F_{4}^{token}$ and $F_{loc}^{token}$ into *s* local feature segments, as follows:

(16) $$\begin{array}{c} Q_{1},\ldots ,Q_{s}=\mathrm{Partition}\left(F_{loc}^{token}\right) \end{array}$$

(17) $$\begin{array}{c} K_{1},\ldots ,K_{s}=\mathrm{Partition}\left(F_{4}^{token}\right) \end{array}$$

(18) $$\begin{array}{c} V_{1},\ldots ,V_{s}=\mathrm{Partition}\left(F_{4}^{token}\right) \end{array}$$

where $F_{loc}^{token}$ and $F_{4}^{token}\in R^{H\times W\times C}$, and s=H×W. Subsequently, we employ a linear layer to transform the token segments into query *Q*, key *K*, and value *V*. The linear projection can be expressed as

(19) $$\begin{array}{c} Q_{i}={\mathrm{Linear}}_{Q}\left(Q_{i}\right),\;K_{i}={\mathrm{Linear}}_{K}\left(K_{i}\right),\;V_{i}={\mathrm{Linear}}_{V}\left(V_{i}\right) \end{array}$$

where i=1,2,…,s. The Linear(*) function denotes a linear projection operator shared among different segments.

To explore the shared information between the $F_{4}$ and $F_{loc}$ features while considering long-range relationships, we first apply Softmax to $K_{j}$ to normalize each element into a probability distribution, and multiply $V_{i}$ (where *i* and *j* range from 1 to *s*). Then, perform element-wise multiplication with *V* to infer the shared feature information between *Q* and *V*. This process can be expressed as

(20) $$\begin{array}{c} {\mathrm{CF}}_{QV}=\frac{\left(V_{1,\ldots ,s}\right)\cdot \mathrm{Softmax}\left({\left(K_{1,\ldots ,s}\right)}^{T}\right)}{\sqrt{d_{k}}}\cdot Q \end{array}$$

where $d_{k}$ is a scaling factor that mitigates the issue of gradient saturation when the dot product increases in the Softmax function. After this, we can easily extract the difference information between *Q* and *V* by removing the shared feature information. This process can be represented as

(21) $$\begin{array}{c} {\mathrm{DF}}_{QV}=\mathrm{Linear}\left(V-{\mathrm{CF}}_{QV}\right) \end{array}$$

To obtain complementary feature information from the $F_{4}$ and $F_{l}oc$ features, we inject the differential features into *Q*, which can be represented as

(22) $$\begin{array}{c} F_{add}={\mathrm{DF}}_{QV}+Q \end{array}$$

Then, we generate $F_{dffm}$ by applying a multi-layer perceptron (MLP) with layer normalization (LN) to $F_{add}$ and adding $F_{add}$ again:

(23) $$\begin{array}{c} F_{dffm}=\mathrm{MLP}\left(\mathrm{LN}\left(F_{add}\right)\right)+F_{add} \end{array}$$

where $F_{dffm}$ is the output of the DFFM.

**Figure 8:**
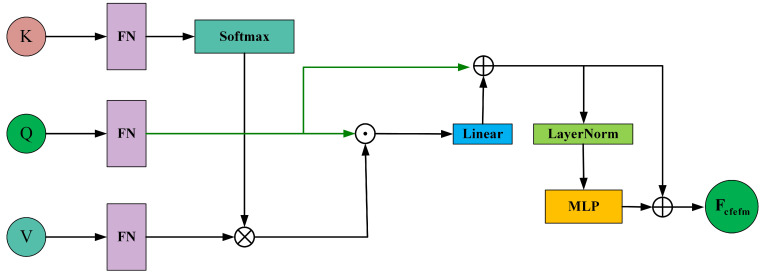
Schematic diagram of the Common Feature Enhancement Fusion Module (CFEFM).

To integrate the shared feature information of $F_{loc}$ into the fused feature and enhance it, the proposed fusion module, after DFFM, adopts a CFEFM to alternately extract common feature information from $F_{loc}$. The structure of CFEFM is shown in Figure 8.

To infuse shared feature information from $F_{loc}$ into the fused information, we first use the segments of $F_{dffm}$ as $Q_{1,\ldots ,s}$ and the segments of $F_{loc}$ as $K_{1,\ldots ,s}$ and $V_{1,\ldots ,s}$. The shared feature information between $F_{dffm}$ and $F_{loc}$ can be expressed as

(24) $$\begin{array}{c} {\mathrm{CM}}_{loc}^{token}=\frac{\left(V_{1,\ldots ,s}\right)\cdot \mathrm{Softmax}\left({\left(K_{1,\ldots ,s}\right)}^{T}\right)}{\sqrt{d_{k}}}\cdot Q \end{array}$$

Next, we add the shared feature information ${\mathrm{CM}}_{loc}^{token}$ to $F_{dffm}$, yielding

(25) $$\begin{array}{c} F_{\mathrm{add}}=\mathrm{Linear}\left({\mathrm{CM}}_{loc}^{token}\right)+Q \end{array}$$

Then, we pass $F_{\mathrm{add}}$ through a multi-layer perceptron (MLP) with layer normalization (LN) to produce

(26) $$\begin{array}{c} F_{\mathrm{cfefm}}=\mathrm{MLP}\left(LN\left(F_{\mathrm{add}}\right)\right)+F_{\mathrm{add}} \end{array}$$

$F_{\mathrm{cfefm}}$ represents the output of the first CFEFM. Subsequently, we infuse the shared feature information between $F_{\mathrm{cfefm}}$ and $F_{loc}$ into the fused feature to enrich it. This process follows the same formulation as Formulas (25) and (26). This design enables the learned features to emphasize the lesion region while mitigating the negative effects caused by noise or artifacts in non-lesion areas of breast tumor ultrasound images. The classification head comprises an average pooling layer and a fully connected layer. It leverages the enriched feature map to predict the probability for each category of the input breast tumor ultrasound image. The classification process can be described as

(27) $$\begin{array}{c} P_{cls}=C\left(F_{fusion},\Theta_{C}\right) \end{array}$$

where *C* is the mapping function of the classifier parameterized by $\Theta_{C}$. $F_{fusion}$ represents the final fused feature, and $P_{cls}$ indicates the probability that the input breast tumor ultrasound image belongs to each category.

#### 2.2.4. Loss Function

In this joint task, the hybrid loss *L* for model training can be described as follows:

(28) $$\begin{array}{c} L=L_{cls}+\lambda L_{loc} \end{array}$$

(29) $$\begin{array}{c} L_{cls}=E\left[-{\bar{Y}}_{cls}^{T}\log\left(P_{cls}\right)\right] \end{array}$$

(30) $$\begin{array}{c} L_{loc}=E\left[-{\bar{Y}}_{pixel}^{T}\log\left(P_{pixel}\right)\right] \end{array}$$

where $L_{cls}$ measures the difference between the true class labels and the class labels predicted by the classifier *C*, and $L_{loc}$ is defined as a lesion region localization loss. λ∈[0,1] is a weighting factor used to adjust the contribution of $L_{loc}$. In $L_{cls}$, $P_{cls}$ represents the probability that the input data *X* belong to class *K*, while ${\bar{Y}}_{cls}$ is a one-hot true label vector with *K* elements. In $L_{loc}$, $P_{pixel}$ represents the probability that each pixel in $F_{mask}$ belongs to a lesion, and ${\bar{Y}}_{pixel}$ is the lesion location label determined by applying a binarization function to $P_{pixel}$.

### 2.3. Hyperparameter Optimization

The deep learning framework selected for this study is PyTorch. The experiments were conducted on a server equipped with two NVIDIA RTX 3090Ti GPUs, an Intel(R) Xeon(R) Gold 6226R CPU, and 128 GB of RAM. The model optimization utilized the adaptive Adam stochastic gradient algorithm [26] to compute the loss function defined in Section 2.2.4. The initial learning rate was set to lr=0.00001, and the model was trained for 200 epochs with a batch size of 8. Input images were preprocessed using data augmentation techniques, including random rotations $\left[90^{\circ },180^{\circ },270^{\circ }\right]$, and random horizontal and vertical flips. The preprocessed images were resized to 256×256 pixels using bilinear interpolation and normalized to the range [0, 1] before being fed into the model. In order to fairly compare the performance of the models, all experiments in this study used the same parameter settings.

In this experiment, we employed ResNet18 and ResNet50 as the backbone networks for our model, resulting in two model versions: ${\mathrm{MFFMT}}^{18}$ and ${\mathrm{MFFMT}}^{50}$. To evaluate training stability, the process was repeated five times, and the mean of each metric was calculated as the final experimental results.

The evaluation metrics used include accuracy, precision, sensitivity, specificity, F1-score, and the area under the ROC curve (AUC).

## 3. Results

### 3.1. Validation of Different Modules’ Effectiveness

**Figure 9:**
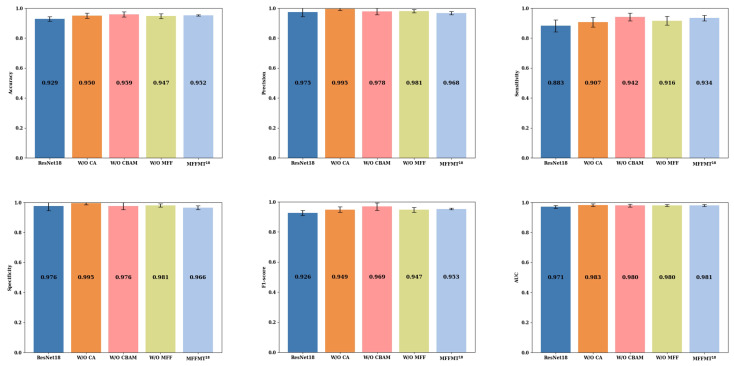
${\mathrm{MFFMT}}^{18}$: The results of ablation experiments on the BUSI dataset.

**Figure 10:**
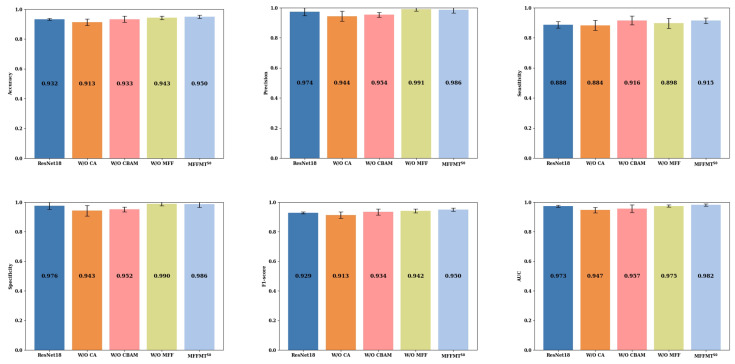
${\mathrm{MFFMT}}^{50}$: The results of ablation experiments on the BUSI dataset.

To investigate the impact of each module on the overall model performance, we removed each module individually, maintaining the parameter settings described in Section 2.3, and retrained the model. Figure 9 and Figure 10 present the ablation study results of our model with ResNet18 and ResNet50 as the backbone networks, respectively, on the BUSI dataset, referred to as ${\mathrm{MFFMT}}^{18}$ and ${\mathrm{MFFMT}}^{50}$.

From Figure 9 and Figure 10, we can observe the performance metrics when MFFMT is stripped of all modules, using ResNet18 and ResNet50 as backbone networks. For ResNet18 and ResNet50, the accuracy is 0.929 and 0.932, precision is 0.975 and 0.974, sensitivity is 0.883 and 0.888, specificity is 0.976 and 0.9756, F1-score is 0.926 and 0.929, and AUC is 0.971 and 0.973 (±0.0081), respectively. When the designed models, ${\mathrm{MFFMT}}^{18}$ and ${\mathrm{MFFMT}}^{50}$, incorporate various modules, there is a noticeable improvement in performance. Accuracy increases by 2.3% (0.952 vs. 0.929) and 1.8% (0.950 vs. 0.932), sensitivity increases by 5.5% (0.938 vs. 0.883) and 2.7% (0.915 vs. 0.888), F1-score increases by 2.7% (0.953 vs. 0.926) and 2.0% (0.949 vs. 0.929), and AUC increases by 1.0% (0.981 vs. 0.971) and 0.9% (0.982 vs. 0.973). However, compared to ResNet18, ${\mathrm{MFFMT}}^{18}$ shows a decrease in precision and specificity by 0.7% (0.968 vs. 0.975) and 1.0% (0.966 vs. 0.976), respectively. On the other hand, compared to ResNet50, ${\mathrm{MFFMT}}^{50}$ shows an improvement in precision and specificity by 1.3% (0.986 vs. 0.974) and 1.0% (0.986 vs. 0.976), respectively. This indicates that a greater number of network layers can lead to better performance.

Additionally, for the ${\mathrm{MFFMT}}^{50}$ model, when the CA module was removed, the model’s accuracy, precision, sensitivity, specificity, F1-score, and AUC decreased by 3.7% (0.913 vs. 0.950), 3.2% (0.954 vs. 0.986), 3.1% (0.884 vs. 0.915), 4.3% (0.943 vs. 0.986), 3.6% (0.913 vs. 0.949), and 3.5% (0.947 vs. 0.982), respectively. This highlights that the Coordinate Attention module, CA, plays a vital role in capturing global information, especially long-range dependencies in breast tumor ultrasound images. Removing the CA module may hinder the model’s ability to leverage global information, potentially affecting the quality of feature representation, and consequently, degrading overall model performance. Similarly, when the CBAM module was removed, the overall performance of the model also suffered a decline. This demonstrates that the spatial attention mechanism in the CBAM module not only helps the model focus on the significance of different positions in the image but also assists in identifying and emphasizing important features across channels. Removing the CBAM module can result in the model ignoring spatial information and failing to effectively utilize channel correlations, thus impacting the classification performance. When the MFF module was removed, the model’s accuracy, sensitivity, F1-score, and AUC decreased by 0.7% (0.943 vs. 0.950), 1.7% (0.898 vs. 0.915), 0.7% (0.942 vs. 0.949), and 0.7% (0.975 vs. 0.982), respectively. In contrast, the precision and specificity increased by 0.5% (0.991 vs. 0.986) and 0.4% (0.990 vs. 0.986). This suggests that the inclusion of the MFF module allows the model to exercise greater caution in identifying potential malignant cases, leading to a tendency to classify suspicious samples as malignant, thus enhancing its capability to detect malignant cases—a crucial aspect in medical diagnostic tasks.

**Figure 11:**
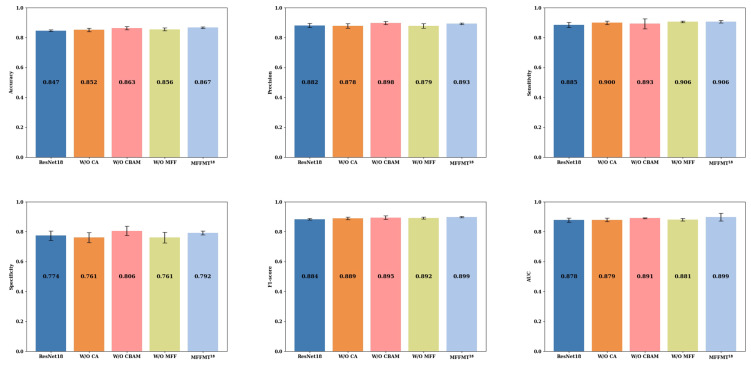
${\mathrm{MFFMT}}^{18}$: The results of ablation experiments on the MIBU dataset.

**Figure 12:**
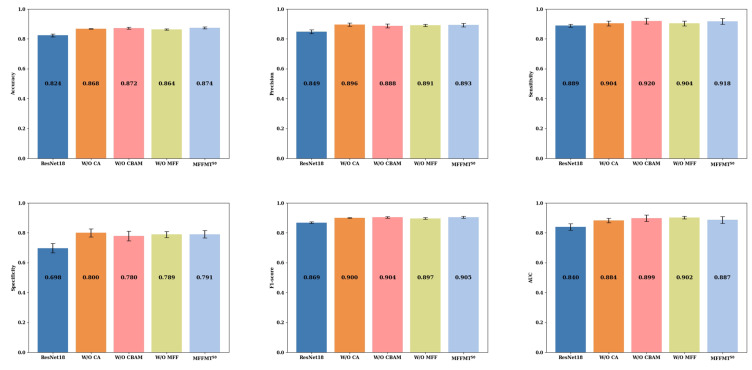
${\mathrm{MFFMT}}^{50}$: The results of ablation experiments on the MIBU dataset.

Figure 11 and Figure 12 illustrate the comparison results of ablation experiments conducted on the MIBU dataset for ${\mathrm{MFFMT}}^{18}$ and ${\mathrm{MFFMT}}^{50}$, respectively. From Figure 11 and Figure 12, it is evident that when MFFMT removes all modules, the accuracy of ResNet18 and ResNet50 is 0.847 and 0.824, the precision is 0.882 and 0.849, the sensitivity is 0.885 and 0.889, the specificity is 0.774 and 0.698, the F1-score is 0.884 and 0.869, and the AUC is 0.878 and 0.840, respectively. On the other hand, when we introduce each module to our designed models ${\mathrm{MFFMT}}^{18}$ and ${\mathrm{MFFMT}}^{50}$, there is a significant performance improvement. The accuracy of ${\mathrm{MFFMT}}^{18}$ and ${\mathrm{MFFMT}}^{50}$ are increased by 2.0% (0.867 vs. 0.847) and 5.0% (0.874 vs. 0.824), the precision by 1.1% (0.893 vs. 0.882) and 4.4% (0.893 vs. 0.849), the sensitivity by 2.1% (0.906 vs. 0.885) and 2.9% (0.918 vs. 0.889), the specificity by 1.8% (0.792 vs. 0.774) and 9.3% (0.791 vs. 0.698), the F1-score by 1.5% (0.899 vs. 0.884) and 3.6% (0.905 vs. 0.869), and the AUC by 0.3% (0.881 vs. 0.878) and 4.7% (0.887 vs. 0.840), respectively. This confirms that the proposed MFFMT model is effective on the MIBU dataset, and particularly the ${\mathrm{MFFMT}}^{50}$ model has a more significant performance improvement.

Additionally, when comparing the results of ${\mathrm{MFFMT}}^{18}$ and ${\mathrm{MFFMT}}^{50}$ on both the BUSI and MIBU datasets, we observe that due to the smaller number of ultrasound images in the BUSI dataset, both the ResNet18 and ResNet50 models tend to overfit. Consequently, when tested on the larger MIBU dataset under the same conditions, the performance of these models significantly declines. In contrast, our proposed MFFMT model effectively mitigates the overfitting phenomenon, especially in the ${\mathrm{MFFMT}}^{50}$ model, where the effect is more pronounced. This suggests that with increased network depth, the likelihood of overfitting rises. However, multi-task learning provides a viable solution, effectively reducing the occurrence of this issue.

### 3.2. Exploring the Impact of Different Feature Fusion Methods on the Model

The proposed MFF module aims to leverage the distinct and shared characteristics present in different feature maps to generate a composite feature map that captures prominent targets and retains rich texture details. To validate its effectiveness, this section compares the module with common fusion methods, namely, Sum and Concat. Sum performs element-wise addition of feature maps while preserving the original channel numbers, whereas Concat merges feature maps by increasing the channel count to facilitate the fusion.

Table 3 and Table 4 present the results of ${\mathrm{MFFMT}}^{18}$ and ${\mathrm{MFFMT}}^{50}$, respectively, using different fusion methods on the BUSI dataset.

From Table 3 and Table 4, it is evident that on the BUSI dataset MFFMT achieves the highest performance with the Multi-Feature Fusion method MFF, reaching optimal levels across all three metrics. Specifically, ${\mathrm{MFFMT}}^{18}$, when utilizing the MFF fusion method, outperforms the next best fusion method, Sum, with increases of 1.4% (0.952 vs. 0.938) in accuracy, 2.7% (0.938 vs. 0.911) in sensitivity, and 1.5% (0.953 vs. 0.938) in F1-score. Additionally, the MFF method’s performance in AUC is only 0.2% (0.981 vs. 0.983) lower than the Sum method. However, ${\mathrm{MFFMT}}^{50}$, when employing the MFF method, exhibits slightly reduced performance compared to the Sum method. This decrease in performance may be attributed to the increased depth of the ${\mathrm{MFFMT}}^{50}$ model, leading to overfitting on the smaller BUSI dataset. Therefore, comparative experiments of different fusion methods were conducted on the larger MIBU dataset, with results presented in Table 5 and Table 6.

From Table 5 and Table 6, it is apparent that MFFMT with the MFF method performs the best. Specifically, ${\mathrm{MFFMT}}^{18}$ with the MFF method exhibits superior results on the MIBU dataset across all metrics when compared to other models. When compared to the next best fusion method, Sum, ${\mathrm{MFFMT}}^{18}$ shows improvements of 1.8% (0.867 vs. 0.849) in accuracy, 1.3% (0.893 vs. 0.880) in precision, 1.3% in sensitivity (0.906 vs. 0.893), 2.6% in specificity (0.792 vs. 0.766), 1.3% in F1-score (0.899 vs. 0.886), and 1.0% (0.881 vs. 0.871) in AUC. Similarly, ${\mathrm{MFFMT}}^{50}$ with the MFF method, when compared to the next best fusion method, Concat, shows improvements of 0.8% (0.874 vs. 0.866) in accuracy, 0.4% (0.893 vs. 0.889) in precision, 0.9% (0.918 vs. 0.913) in sensitivity, 0.7% (0.791 vs. 0.784) in specificity, and 0.7% (0.906 vs. 0.899) in F1-score. The only slight decrease in performance was observed in the AUC metric, where ${\mathrm{MFFMT}}^{50}$ achieved 0.1% (0.887 vs. 0.888) lower than the Concat method. In summary, our proposed MFF method is effective in leveraging features that contribute to the classification of benign and malignant breast tumors. Additionally, it is evident that deeper networks benefit more from feature fusion on the MIBU dataset, suggesting that when features are fused at a shallower level they might contain more redundant information and noise. On the other hand, deeper feature fusion helps to filter out redundant information and noise, resulting in more discriminative and representative features. Moreover, by fusing features at deeper layers, the network can learn more complex feature interactions and relationships, enhancing the network’s expressive capabilities and classification performance.

### 3.3. Exploring the Impact of Loss Function Parameters on Model Performance

**Figure 13:**
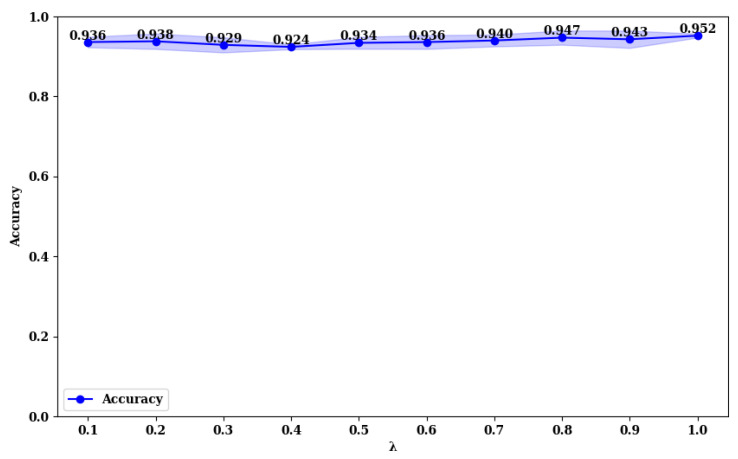
Accuracy rate of ${\mathrm{MFFMT}}^{18}$ with different λ values on the BUSI dataset.

**Figure 14:**
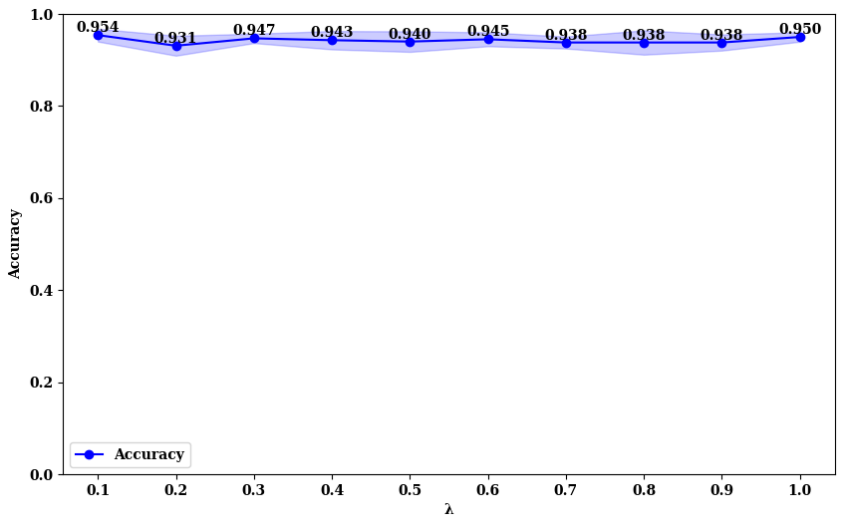
Accuracy rate of ${\mathrm{MFFMT}}^{50}$ with different λ values on the BUSI dataset.

**Figure 15:**
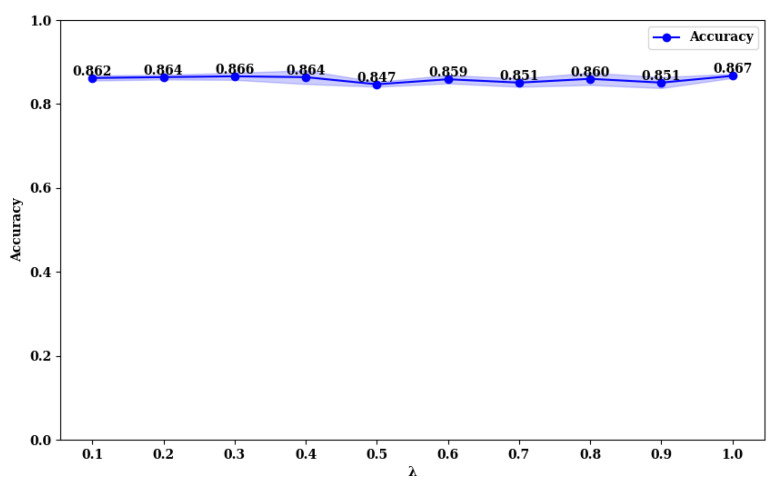
Accuracy rate of ${\mathrm{MFFMT}}^{18}$ with different λ values on the MIBU dataset.

**Figure 16:**
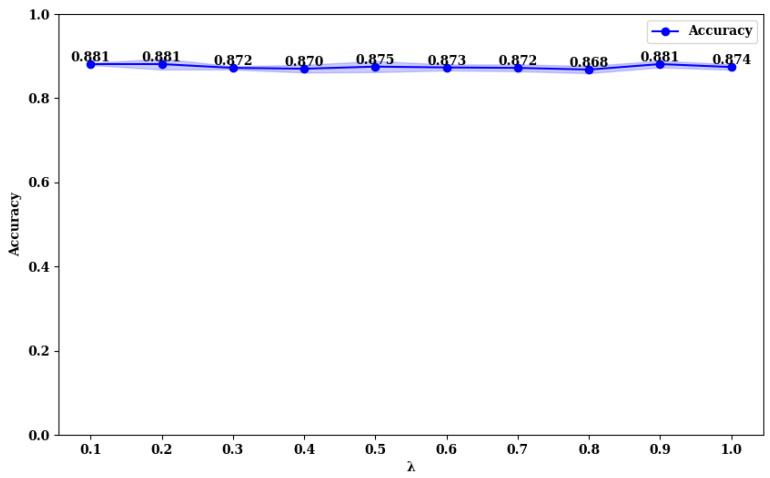
Accuracy rate of ${\mathrm{MFFMT}}^{50}$ with different λ values on the MIBU dataset.

To explore the impact of the λ value in the loss function (Equation (28)), we compared the accuracy of ${\mathrm{MFFMT}}^{18}$ and ${\mathrm{MFFMT}}^{50}$ across the BUSI and MIBU datasets, using λ values in the range (0, 1). The results are presented in Figure 13, Figure 14, Figure 15 and Figure 16.

From Figure 13 and Figure 14, it is evident that on the BUSI dataset, the ${\mathrm{MFFMT}}^{18}$ model achieves its highest accuracy at a λ value of 1.0, with an accuracy of 0.952. At this point, the model’s stability (minimum standard deviation) is also optimal. For the ${\mathrm{MFFMT}}^{50}$ model, the highest accuracy is achieved at a λ value of 0.1, with an accuracy of 0.954, and it reaches the second-best performance at a λ value of 1.0, with an accuracy of 0.950. This phenomenon is consistent on the MIBU dataset as well. This indicates that in the multi-task learning framework for breast tumor image lesion localization and classification, the ${\mathrm{MFFMT}}^{18}$ model, which uses ResNet18 as the backbone network, achieves the best classification performance when the weights for lesion localization loss and classification loss are equal (λ=1). This can be attributed to the shallower nature of ResNet18, which has fewer parameters and limited feature extraction capabilities. The synergistic effect between the classification and lesion localization tasks is stronger, allowing balanced losses to better leverage shared features, thereby enhancing classification performance. Additionally, the gradient propagation in shallower networks is more stable, allowing simultaneous optimization of both tasks’ losses. Conversely, for the ${\mathrm{MFFMT}}^{50}$ model, which uses ResNet50 as the backbone network, the best classification performance is observed when the weight for the lesion localization loss is lower (λ=0.1). This is because ResNet50, being deeper with more parameters, has stronger feature extraction capabilities and can learn more fine-grained classification features. A higher weight for the lesion localization loss might interfere with the learning of classification features and cause instability in the optimization process. By reducing the weight of the localization loss, the interference with classification features is minimized, enhancing the stability of the optimization process, and thus, improving classification performance.

### 3.4. Comparison with Other Models

In this study, we implemented and compared the proposed method with two state-of-the-art MTL methods and four single-task classification methods. The four single-task classification methods include ResNet18 [27], ResNet50 [27], EfficientNet [28], and Vision Transformer (ViT) [29]. Additionally, we evaluated two recently proposed MTL methods for breast tumor classification: RMTL [12] and LA-Net [30]. RMTL is a typical shared-backbone MTL method specifically designed for breast tumor imaging, similar to our approach. LA-Net is also a joint localization and malignancy classification model for breast tumors, which can utilize various CNN-based networks as its feature extraction backbone. In our experimental setup, we used ResNet50 and ResNet18 as the feature extraction backbones for our method, RMTL, and LA-Net, resulting in the models being named ${\mathrm{MFFMT}}^{18}$, ${\mathrm{MFFMT}}^{50}$, ${\mathrm{LA}-\mathrm{Net}}^{18}$, ${\mathrm{LA}-\mathrm{Net}}^{50}$, ${\mathrm{RMTL}}^{18}$, and ${\mathrm{RMTL}}^{50}$, respectively. Table 7 and Table 8 present the comparative results of our model against the other models on the BUSI and MIBU datasets.

From Table 7, we can observe that among the single-task models, EfficientNet performs the best, achieving an accuracy, precision, sensitivity, specificity, F1-score, and AUC of 0.956, 0.980, 0.932, 0.955, and 0.984, respectively. Our ${\mathrm{MFFMT}}^{50}$ model achieves the second-best performance, only falling short of EfficientNet by 0.6% in accuracy (0.950 vs. 0.956), 1.7% in sensitivity (0.915 vs. 0.932), 0.6% in F1-score (0.949 vs. 0.955), and 0.2% in AUC (0.982 vs. 0.984). However, it surpasses EfficientNet in precision and specificity by 0.6% (0.986 vs. 0.980) and 0.5% (0.986 vs. 0.981), respectively. These results may be attributed to EfficientNet’s deep architecture (237 layers), which allows it to learn more complex and abstract feature representations. Such representations can better capture high-level patterns and structures in the data, thus enhancing model performance. However, this depth also increases the risk of overfitting, particularly when the training data are limited. The enhanced expressive power of the model makes it more prone to memorizing details and noise in the training data, rather than generalizing the true underlying patterns in the data.

Additionally, as observed in Table 7, our designed model achieves the best performance when compared to other multi-task models. Specifically, compared to the RMTL model, ${\mathrm{MFFMT}}^{50}$ outperforms ${\mathrm{RMTL}}^{50}$ in terms of accuracy, precision, sensitivity, specificity, F1-score, and AUC by 1.6% (0.950 vs. 0.934), 1.7% (0.986 vs. 0.969), 1.3% (0.915 vs. 0.902), 1.9% (0.986 vs. 0.967), 1.6% (0.949 vs. 0.933), and 1.4% (0.982 vs. 0.968), respectively. This indicates that MFFMT is more effective in capturing the latent features in lesion perception tasks that aid in the benign and malignant classification of breast tumors while alleviating potential information-sharing conflicts during model training. Moreover, we notice that the ViT model exhibits relatively lower accuracy. This suggests that traditional convolutional neural networks (CNNs) are still better suited for handling image data, especially on smaller datasets, compared to pure Transformer models.

As shown in Table 8, when the models are trained on the MIBU dataset, our designed model MFFMT demonstrates the best overall performance. Specifically, the ${\mathrm{MFFMT}}^{50}$ model achieves an accuracy of 0.874, precision of 0.893, sensitivity of 0.918, specificity of 0.791, F1-score of 0.906, and AUC of 0.887. Interestingly, by comparing the results from Table 7 and Table 8, we can see that although EfficientNet performed exceptionally well on the BUSI dataset, its performance on the MIBU dataset was significantly worse. This could be because the MIBU dataset was collected using different ultrasound devices, leading to notable differences in image brightness, texture, background, or object angles, which resulted in poorer robustness for EfficientNet. In contrast, the MFFMT model demonstrated better robustness. Moreover, by comparing the precision, sensitivity, and specificity results of the MFFMT model on the BUSI and MIBU datasets, we can easily observe that on the BUSI dataset, where the number of benign tumors greatly exceeds that of malignant tumors, the model’s ability to predict benign tumors is higher than its ability to predict malignant tumors. Conversely, on the MIBU dataset, where the number of malignant tumors far exceeds that of benign tumors, the model is more sensitive to identifying malignant tumors. This phenomenon underscores the importance of dataset balance when training machine learning models. If a dataset significantly over-represents one class, the model may become biased towards predicting the more frequent class. In clinical settings, different types of tumors may have varying frequencies in different environments. Therefore, the model’s performance may vary in different clinical environments. Understanding and accounting for these environmental differences is crucial for the successful application of models in clinical practice.

### 3.5. Explainable Analysis

**Figure 17:**
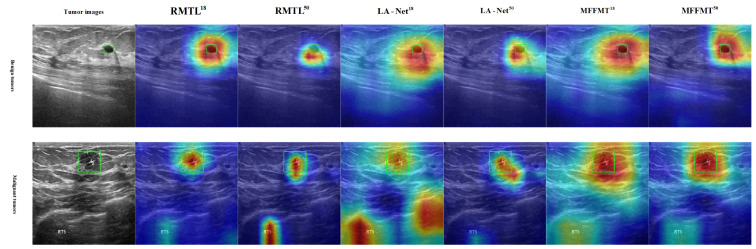
Example of Grad-CAM heatmap on the BUSI dataset.

**Figure 18:**
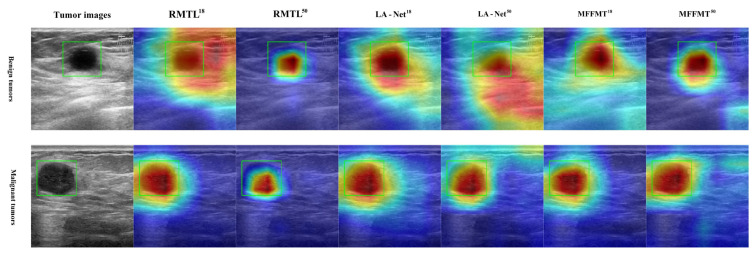
Example of Grad-CAM heatmap on the MIBU dataset.

Class activation mapping (CAM) [31,32] has been widely used to interpret classification networks across various applications, including breast ultrasound image analysis [30]. This technique enhances the interpretability of model performance by visualizing the regions that contribute most to classification decisions. Gradient-weighted class activation mapping (Grad-CAM) [32] is an effective and widely applicable CAM technique that highlights potential regions of interest (ROIs) based on the gradient scores of each class. In this study, Grad-CAM is utilized to identify discriminative lesion regions in breast ultrasound images. Our proposed method, along with other multi-task learning methods, underwent Grad-CAM analysis. Examples of the generated Grad-CAM visualizations are shown in Figure 17 and Figure 18. It was observed that the attention regions identified by our method exhibited a higher degree of overlap with the actual lesion locations. These examples indicate that MFFMT effectively assists the feature extraction process, enabling the feature extractor to capture more discriminative information from the lesion regions. Additionally, by analyzing the visualizations across all data, we found that the MFFMT model produced a higher number of effective visual results compared to other models. Consequently, our framework reduces interference from noisy backgrounds and enhances overall classification performance.

## 4. Conclusions and Future Directions

In breast imaging analysis, MTL methods are widely used for lesion region perception and classification, aiding in breast cancer diagnosis and treatment. However, traditional MTL methods often overlook how to effectively utilize knowledge from auxiliary tasks for tumor classification in their shared-backbone network architectures, which may lead to information-sharing conflicts and impact the performance of the main task. Additionally, these models have limitations in extracting fine-grained tumor features.

This study proposes a Multi-Feature Fusion Multi-Task convolutional neural network model for joint lesion perception and benign–malignant classification of breast tumors. The model effectively extracts richer feature information for lesion region perception and integrates it with semantic feature information of the tumor. This approach addresses the inherent information-sharing conflict issues in shared-backbone multi-task learning methods and the overfitting problems faced by single-task models, thereby improving the performance of benign–malignant classification of breast tumors. The experimental results show that the designed MFFMT model achieves excellent results through Multi-Feature Fusion and demonstrates good robustness under different data distributions. Moreover, we used visualization techniques for interpretability analysis of the model, making the prediction process more transparent and understandable. This enhances trust in the model by allowing users to understand how the model makes predictions or decisions based on input data, rather than passively accepting the model’s output.

Despite the good performance of our model, there are still some limitations. For example, our model was trained under a fully supervised learning paradigm, which requires a large amount of data with category labels and pixel-level tumor segmentation annotations or bounding boxes, with it often being both time-consuming and labor intensive to obtain the latter. Semi-supervised learning (SSL) techniques have received increasing attention for training deep learning models by utilizing unlabeled data and reducing the need for large amounts of labeled data. Future research could explore various approaches to address this issue. For instance, investigating new semi-supervised learning techniques and designing models suitable for breast tumor classification using SSL could be valuable. This might include experimenting with generative adversarial networks (GANs) or self-supervised learning methods to leverage unlabeled data and improve model performance. Additionally, employing data augmentation and pseudo-labeling methods to develop new strategies for enhancing training data diversity and improving model generalization could be beneficial. This includes generating synthetic images and annotations or using self-training methods to enhance the model’s adaptability to unlabeled data. Finally, integrating multi-task learning with semi-supervised learning could enhance the model’s performance on limited labeled data by combining relevant auxiliary tasks (such as region segmentation or anomaly detection) with the main task of breast tumor classification.

In addition, our MFFMT model incorporates multi-feature fusion and multi-task learning to enhance breast tumor classification performance. However, this complex network architecture can lead to higher computational complexity compared to single-task models. The MFFMT model includes multiple convolutional layers and feature fusion modules, which result in significant computational and parameter demands, requiring substantial computational resources during both training and inference. In the future, exploring model pruning and quantization techniques could reduce the number of parameters and computational load, thereby accelerating inference speed. Furthermore, in clinical applications, fast and accurate inference is crucial. After training, the MFFMT model’s inference time primarily comes from model loading. Therefore, developing a lightweight version of the model in the future to reduce inference time and improve the feasibility of real-time applications is an important step. Lastly, in practice, different medical institutions use various ultrasound imaging devices, which can lead to inconsistencies in data distribution and affect model performance robustness. To address this issue, future research could incorporate domain adaptation techniques from transfer learning to effectively handle potential problems arising from discrepancies between datasets.

## Figures and Tables

**Table 1:** Details of the BUSI dataset.

Category	Training Set	Test Set
Malignant tumors	176	45
Benign tumors	407	42

**Table 2:** Details of the MIBUS dataset.

Category	Training Set	Test Set
Malignant tumors	1221	337
Benign tumors	787	177

**Table 3:** Results of ${\mathrm{MFFMT}}^{18}$ on the BUSI dataset using different fusion methods.

Fusion Method	Accuracy	Precision	Sensitivity	Specificity	F1-Score	AUC
Sum	0.938	0.967	0.911	0.966	0.938	**0.983**
Concat	0.933	**0.971**	0.898	**0.971**	0.933	0.979
MFF (ours)	**0.952**	0.968	**0.938**	0.966	**0.953**	0.981

**Table 4:** Results of ${\mathrm{MFFMT}}^{50}$ on the BUSI dataset using different fusion methods.

Fusion Method	Accuracy	Precision	Sensitivity	Specificity	F1-Score	AUC
Sum	**0.952**	0.977	0.929	0.976	**0.952**	0.981
Concat	0.950	0.956	**0.947**	0.952	0.951	0.980
MFF (ours)	0.950	**0.986**	0.915	**0.986**	0.949	**0.982**

**Table 5:** Results of ${\mathrm{MFFMT}}^{18}$ on the MIBU dataset using different fusion methods.

Fusion Method	Accuracy	Precision	Sensitivity	Specificity	F1-Score	AUC
Sum	0.849	0.880	0.893	0.766	0.886	0.871
Concat	0.849	0.871	0.904	0.744	0.887	0.863
MFF (ours)	**0.867**	**0.893**	**0.906**	**0.792**	**0.899**	**0.881**

**Table 6:** Results of ${\mathrm{MFFMT}}^{50}$ on the MIBU dataset using different fusion methods.

Fusion Method	Accuracy	Precision	Sensitivity	Specificity	F1-Score	AUC
Sum	0.863	0.882	0.913	0.767	0.897	0.873
Concat	0.866	0.889	0.909	0.784	0.899	**0.888**
MFF (ours)	**0.874**	**0.893**	**0.918**	**0.791**	**0.906**	0.887

**Table 7:** Comparison results of various models in the BUSI dataset.

Model Method	Accuracy	Precision	Sensitivity	Specificity	F1-Score	AUC
ResNet18	0.929	0.975	0.883	0.976	0.926	0.971
ResNet50	0.932	0.974	0.888	0.976	0.929	0.973
EfficientNet	**0.956**	0.980	**0.932**	0.981	**0.955**	**0.984**
VIT	0.788	0.798	0.776	0.800	0.784	0.839
${\mathrm{RMTL}}^{18}$	0.924	0.977	0.876	0.976	0.923	0.962
${\mathrm{RMTL}}^{50}$	0.934	0.969	0.902	0.967	0.933	0.968
${\mathrm{LA}-\mathrm{Net}}^{18}$	0.920	0.968	0.876	0.967	0.918	0.960
${\mathrm{LA}-\mathrm{Net}}^{50}$	0.913	0.956	0.871	0.957	0.912	0.964
${\mathrm{MFFMT}}^{18}$	0.952	0.968	0.938	0.966	0.953	0.981
${\mathrm{MFFMT}}^{50}$	0.950	**0.986**	0.915	**0.986**	0.949	0.982

**Table 8:** Comparison results of various models on the MIBU dataset.

Model Method	Accuracy	Precision	Sensitivity	Specificity	F1-Score	AUC
ResNet18	0.847	0.882	0.885	0.774	0.884	0.878
ResNet50	0.824	0.849	0.889	0.698	0.869	0.840
EfficientNet	0.866	0.888	0.911	0.780	0.899	0.880
VIT	0.835	0.863	0.893	0.724	0.876	0.798
${\mathrm{RMTL}}^{18}$	0.839	0.877	0.879	0.764	0.877	0.853
${\mathrm{RMTL}}^{50}$	0.816	0.845	0.882	0.691	0.863	0.838
${\mathrm{LA}-\mathrm{Net}}^{18}$	0.834	0.868	0.881	0.745	0.874	0.860
${\mathrm{LA}-\mathrm{Net}}^{50}$	0.860	0.875	0.917	0.750	0.895	**0.887**
${\mathrm{MFFMT}}^{18}$	0.867	**0.893**	0.906	**0.792**	0.899	0.881
${\mathrm{MFFMT}}^{50}$	**0.874**	**0.893**	**0.918**	0.791	**0.906**	**0.887**

## Data Availability

In this research, the breast tumor ultrasound image datasets we used all come from public datasets. The BUSI dataset can be downloaded from [33]. And the MIBUS dataset can be obtained from [34]. All source code of our experiments will be released at https://github.com/jinzhuwei/MFFMT (accessed on 8 May 2012).

## Abbreviations

The following abbreviations are used in this manuscript:

MTL	Multi-task learning
MFFMT	Multi-Feature Fusion Multi-Task
MFF	Multi-Feature Fusion
CLEP	Contextual Lesion Enhancement Perception
CAD	Computer-aided diagnosis
BUSI	Breast Ultrasound Image
CBAM	Convolutional Block Attention Module
SAM	Spatial Attention Module
CA	Coordinate Attention
MLP	Multi-layer perceptron
BN	Batch normalization
PE	Patch Embedding
DFFM	Discrepancy Feature Fusion Module
CFEFM	Common Feature Enhancement Fusion Module
CAM	Class activation mapping
SSL	Semi-supervised learning
